# Finding Candidate Drugs for Hepatitis C Based on Chemical-Chemical and Chemical-Protein Interactions

**DOI:** 10.1371/journal.pone.0107767

**Published:** 2014-09-16

**Authors:** Lei Chen, Jing Lu, Tao Huang, Jun Yin, Lai Wei, Yu-Dong Cai

**Affiliations:** 1 College of Information Engineering, Shanghai Maritime University, Shanghai, People's Republic of China; 2 Department of Medicinal Chemistry, School of Pharmacy, Yantai University, Shandong, Yantai, People's Republic of China; 3 Department of Genetics and Genomic Sciences, Icahn School of Medicine at Mount Sinai, New York, New York, United States of America; 4 Institute of Systems Biology, Shanghai University, Shanghai, People's Republic of China; Singapore Immunology Network, Agency for Science, Technology and Research (A*STAR), Singapore

## Abstract

Hepatitis C virus (HCV) is an infectious virus that can cause serious illnesses. Only a few drugs have been reported to effectively treat hepatitis C. To have greater diversity in drug choice and better treatment options, it is necessary to develop more drugs to treat the infection. However, it is time-consuming and expensive to discover candidate drugs using experimental methods, and computational methods may complement experimental approaches as a preliminary filtering process. This type of approach was proposed by using known chemical-chemical interactions to extract interactive compounds with three known drug compounds of HCV, and the probabilities of these drug compounds being able to treat hepatitis C were calculated using chemical-protein interactions between the interactive compounds and HCV target genes. Moreover, the randomization test and expectation-maximization (EM) algorithm were both employed to exclude false discoveries. Analysis of the selected compounds, including acyclovir and ganciclovir, indicated that some of these compounds had potential to treat the HCV. Hopefully, this proposed method could provide new insights into the discovery of candidate drugs for the treatment of HCV and other diseases.

## Introduction

Hepatitis C virus (HCV) infection is a major cause of certain chronic liver-related diseases, including cirrhosis, liver failure, and hepatocellular carcinoma, and it affects 170 million people worldwide [1]. A classic and widely used treatment for HCV is ribavirin combined with pegylated interferon alpha (PegIFN-α). The efficacy of this combination is limited for the genotype 1 virus, which is the most common type in humans. In 2011, two direct-acting antiviral drugs (DAAs), telaprevir and boceprevir, were approved by the FDA for the treatment of HCV [2], [3]. In addition, the combination of DAAs, ribavirin, and PegIFN-α showed high sustained virological response rates in genotype 1 HCV patients and thus became a standard care of regimen for such patients [4].

It is well known that the research and development of a drug requires comprehensive experimental testing, which often costs millions of dollars, involves several thousand animals, and takes many years to complete. In contrast, only a handful of chemicals have met the regulatory requirements for drug approval. Thus, it is very attractive to develop quick, reliable, and *in silico* methods, *e.g.*, using structure-activity relationships (SARs), to predict the anti-HCV activities of chemicals [5], [6], [7].

Most previous SAR models of HCV inhibitors are developed based on molecular descriptors and machine learning methods. Weidlich *et al.* developed a model using random forest and *k* nearest neighbor simulated annealing as machine learning classifiers and 2048-bit Morgan fingerprints of radius 2 as features to measure the genotype 1b HCV inhibition activity of 679 compounds [8]. Wang *et al.* built models using multi-linear regression, support vector machine and molecular descriptors for HCV NS5B inhibitors [9]. Speck-Planche *et al.* summarized some of the anti-HCV prediction models using structure- and ligand-based computational-aided drug design methods. Moreover, an anti-HCV model was introduced using atom-centered fragments, functional group counts, and spectral moments of the bond adjacency matrix as features [10]. The above-mentioned models were generated using the structures of chemicals and proteins. In our study, we attempted to employ the information of chemical-chemical and chemical-protein interactions, including information concerning not only structures, complex network and molecular activities that can be categorized as “direct interaction” [11] but also biology pathways and proteins/chemicals function relationships that are referred to as “indirect interaction” [11], to discover candidate drugs for HCV. It has been reported that interactive compounds often share similar functions [12], [13], [14], [15], [16], thus implicating that interactive compounds of known drugs for HCV may also have the capability to treat hepatitis C. The potential compounds were further evaluated and filtered based on various scores for the interactions observed between them and the target genes of HCV or three approved HCV drugs. An analysis of the final obtained compounds suggested that some of these compounds have the potential to treat HCV.

To predict the anti-HCV activity of chemicals, another aim of a potential prediction model is to provide useful information for drug repositioning. Our previous studies verified that some drugs were effective to the ‘wrong’ indications, which subsequently helped reposition drugs to their new indications [12]. Thus, in this study, according to the assumption that interactive drugs are more likely to share the same properties, we investigated the possibility of repositioning the interactive compounds of the three approved drugs by retrieving the related references and thereby attempted to propose treatments as alternatives to the three drugs.

## Materials and Methods

### Drugs and target genes of HCV

Three approved drugs, ribavirin (Pubchem ID: CID000037542), telaprevir (Pubchem ID: CID003010818), and boceprevir (Pubchem ID: CID010324367), were reported as anti-HCV drugs in Drugbank (http://www.drugbank.ca/) [17], [18], a well-known bioinformatics and cheminformatics resource that combines detailed drug information with comprehensive drug targets. In addition, 421 target genes were retrieved from De Chassey *et al.*'s study [19], which was also used in Huang *et al.*'s study [20]. The names of these genes are listed in Table S1.

### Chemical-chemical and chemical-protein interactions

Recently, interactive compounds were reported to possibly share more highly similar functions than non-interactive ones [12], [13], [14], [15], [16]. It can be inferred that the interactive compounds of the three drugs mentioned in Section “Drugs and target genes of HCV” may be more closely related to HCV. To define the interactivity of compounds, we downloaded the information detailing chemical-chemical interactions from STITCH (Search Tool for Interactions of Chemicals) [11] (http://stitch.embl.de/,chemical_chemical.links.detailed.v3.1.tsv.gz), a well-known database containing both the known and predicted interactions of chemicals and proteins. In the obtained file, each chemical-chemical interaction consists of two compounds and five scores: (1) Similarity; (2) Experimental; (3) Database; (4) Textmining; (5) Combined_score. In detail, the scores of Similarity, Experimental and Database were obtained according to chemical structures, activities and reactions, respectively; the score of Textmining was calculated based on chemical co-occurrence in literatures, and the score of Combined_score was obtained by integrating the aforementioned four scores [11]. To note the interactivity of compounds, we adopted the Combined_score to define interactive compounds, *i.e.*, two compounds were interactive if and only if their Combined_score was greater than zero. Interactive compounds may have similar structures, activities, or reactions, thereby suggesting that they have the potential to be in the same pathways. Thus, the interactive compounds of the three HCV drugs may be involved in pathways that are related to HCV. Therefore, it is reasonable to investigate the anti-HCV activity of the interactive compounds of the three approved drugs. In addition, these five scores were also used to cluster the interactive compounds of the three approved drugs (see Section “Further selection of candidate compounds using a clustering algorithm”), thereby assisting in the discovery of candidate drug compounds for HCV. For the latter formulation, the score of Similarity, Experimental, Database, Textmining and Combined_score of the interaction between compounds *c*_1_ and *c*_2_ are denoted as follows: 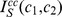, 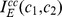, 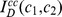, 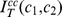 and 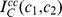, respectively.

A general way to discover candidate drugs for the treatment of HCV is to analyze the relationship between the candidate drugs and the targeted HCV genes. Thus, the information detailing chemical-protein interactions was employed to evaluate the candidate drugs extracted by chemical-chemical interactions. This information was also retrieved from STITCH (protein_chemical.links.detailed.v3.1.tsv.gz) [11]. Similarly, each interaction in the obtained file contains one compound, one protein, and four scores: (1) Experimental; (2) Database; (3) Textmining; (4) Combined_score. Because Combined_score integrates the other three scores, we used the Combined_score to indicate the strength of the interaction between one compound and one protein. Similar to the five scores for the chemical-chemical interactions, all four scores were used to cluster the interactive compounds of the three approved drugs (see Section “Further selection of candidate compounds using a clustering algorithm”). Thus, the scores for Experimental, Database, Textmining and Combined_score of the interaction between compound *c* and protein *p* were denoted as 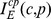, 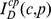, 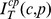 and 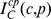, respectively.

### Method used to find candidate compounds

The main idea behind this method is based on the fact that the interactive compounds often share similar functions [12], [13], [14], [15], [16]. Therefore, we focused on investigating the interactive compounds of the three reported drugs for HCV. The detailed procedures of the method were described as follows:

1. The information detailing chemical-chemical interactions and retrieved from STITCH was used to extract the interactive compounds of three drugs for HCV. These compounds and the three drugs comprised a compound set denoted by *D_IC_*.
2. Extract the chemical-protein interactions such that the compounds were in *D_IC_* and the proteins were the target genes of HCV.
3. For each compound *c* in *D_IC_*, let 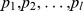 be the target genes of HCV such that 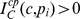 for 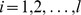. Calculate its determination value, defined as the mean value of 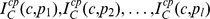.

Higher determination values for a compound indicate that it has a strong association with the target genes of HCV, further suggesting that it is more likely to be able to treat HCV. However, a drug compound with a determination value of 0 is least likely to have the ability to treat HCV. Accordingly, we selected drug compounds with a determination value greater than 0 as candidate compounds.

### Randomization test

As mentioned in Section “Method used to find candidate compounds”, by using this approach, a set of candidate drug compounds can be obtained. However, some of these drug compounds may have a special relationship with human genes, which may result in high determination value, even if we randomly select some human genes. To avoid these false discoveries and encourage future studies on discovering novel genes for some diseases [21], [22], the following randomization test was conducted to further evaluate the candidate drug compounds:

1. Randomly selected 1,000 gene sets, *e.g.*, 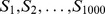, with the same size of known target genes of HCV;
2. For each *S_i_*, execute the method described in Section “Method used to find candidate compounds” by replacing the target genes of HCV with genes in *S_i_*;
3. For each candidate compound *c* obtained in Section “Method used to find candidate compounds”, calculate its p-value using
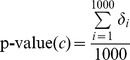
(1)where *δ_i_* was set to be 1 if the determination value of *c* on *S_i_* was greater than that of *c* on the set containing target HCV genes.

It is straightforward to conclude that a smaller p-value for a candidate drug compound suggests that the drug is more likely to have anti-HCV ability. To exclude false discoveries, the p-values of the approved HCV drugs were also computed. Additionally, we selected the p-value of one approved drug as the threshold to filter candidate compounds, *i.e.*, candidate compounds with p-values no more than the threshold were retained for further selection.

### Further selection of candidate compounds using a clustering algorithm

Based on the above two steps, a set of candidate compounds was obtained. However, some of these may still be false discoveries, particularly in cases when many candidate compounds remain. Thus, it is necessary to continue further selection.

According to Sections “Method used to find candidate compounds” and “Randomization test”, we used only the Combined_score for both chemical-chemical and chemical-protein interactions. Although the Combined_score for chemical-chemical interactions includes the Similarity, Experimental, Database, Textmining scores and the Combined_score for chemical-protein interactions includes the Experimental, Database, Textmining scores, *i.e.*, these scores were indirectly used in the above two steps, the direct application of all of these scores may help us more effectively exclude false discoveries. Thus, the five scores of chemical-chemical interactions and the four scores of chemical-protein interactions were used to extract features for each of the candidate compounds remaining after the randomization test, and the EM (expectation maximization) algorithm was employed to cluster these candidate compounds.

#### Feature construction

For each candidate compound *c*, the five chemical-chemical interaction scores induced five features. Because these features were extracted in a similar process, we here only describe how to extract features using Similarity. Let 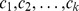 be the approved drugs for HCV such that 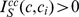 for 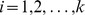 (In fact, *k*≤3 in this study). Take the mean value of 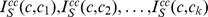 as the feature for Similarity. In particular, this feature was set to be zero if *k* = 0. Similarly, the four chemical-protein interaction scores induced four features for each candidate compound *c*. Here, we only provide a description of how to extract features using Experimental; the other features can be extracted in a similar process. Let 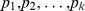 be the target genes of HCV such that 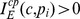 for 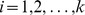. Take the mean value of 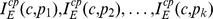 as the feature for Experimental. This feature was set to be zero if *k* = 0. Finally, based on both sets of scores, each candidate compound was represented by nine features.

#### EM algorithm

The Expectation-maximization (EM) algorithm was first proposed by Dempster *et al.* [23]. It is an iterative method for finding the maximum likelihood of parameters in statistical models, where the model depends on unobserved latent variables. The EM iteration alternates between performing an expectation (E) step and a maximization (M) step. The EM algorithm can be defined as follows:

Input: an observed data set *Y*, an unobserved latent data set *Z*, the joint distribution 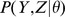 and the conditional distribution 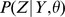


Output: parameter 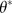

1. Select an initial parameter 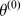
and set *i* = 0, begin iteration
2. E-step: Suppose that 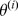 is the current estimated value of parameter *θ*; compute the following objective function:
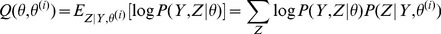
(2)
3. M-step: Find the new estimated value of parameter *θ* (*i.e.*, 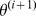) by maximizing 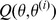:
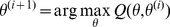
(3)
4. Check the convergence condition 
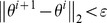
. If the convergence condition is not met, *i* = *i*+1, go to (II); else 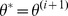.

If we suppose that the data set is drawn from a distribution that can be approximated using a mixture of Gaussian distributions, the EM algorithm can be applied to clustering. Now, the unobserved data set *Z* represents which Gaussian the datum in observed data set *Y* comes from. By utilizing the EM algorithm, we are able to estimate the parameters of each Gaussian and assign each datum to a particular one.

Weka (Waikato Environment for Knowledge Analysis) [24], developed by the University of Waikato in New Zealand, is a popular suit of machine learning software. Many state-of-the-art machine learning algorithms and data preprocessing tools are integrated in this software. A tool termed ‘EM’ in Weka implements the EM algorithm. Thus, it was employed in this study to cluster the candidate compounds that remained following the randomization test. For convenience, this program was run with its default configuration. In this configuration, it can automatically select the class number of clustering via cross-validation.

## Results

### Results of the method

Based on the information detailing chemical-chemical interactions that was retrieved from STITCH, we obtained 623 compounds that contained three drugs and their interactive compounds, *i.e.*, *D_IC_* contained 623 compounds. These compounds and the 421 target genes were used to obtain 955 chemical-protein interactions, which are available in Table S2. These interactions involved 272 compounds and 235 proteins (counted by Ensembl IDs). It is necessary to note that two of the three drugs (CID000037542 and CID003010818) were members of the 272 compounds, while the other drug (CID010324367) was not included in these interactions. According to the method, we calculated the determination value for each compound in *D_IC_*. If one compound in *D_IC_* was not involved in any chemical-protein interaction, its determination value was set to be zero. Finally, we obtained 272 compounds with determination values greater than zero. These 272 compounds and their determination values are provided in Table S3; the determination values of the drugs CID000037542 and CID003010818 were 504.27 and 338, respectively. Accordingly, we took 270 compounds, aside from CID000037542 and CID003010818, as candidate drug compounds for HCV.

### Results of the randomization test

The randomization test was conducted to further evaluate the 270 candidate drug compounds. The p-values of these drug compounds, CID000037542 and CID003010818 were obtained and are listed in Table S3. The p-values of drugs CID000037542 and CID003010818 were 0.269 and 0.298, respectively. The p-value of drug CID010324367 was not computed because its determination value was zero. For a wide selection, the p-value of drug CID003010818 was set as the threshold for filtering the 270 compounds obtained in Section “Results of the method”, *i.e.*, compounds with p-values equal to or less than 0.298 were selected for further analysis, which resulted in 137 candidate drug compounds, including the approved drugs CID000037542 and CID003010818.

### Results of the clustering algorithm

The remaining 137 candidate drug compounds, including the approved drugs CID000037542 and CID003010818, were represented by nine features according to Section “Further selection of candidate compounds using a clustering algorithm”. We only used seven of these features to represent the drug compounds because the values for the chemical-chemical interaction features Experimental and Database were all zero. Next, ‘EM’ in Weka was used to cluster these drug compounds. The clustering results are available in Table S4, where we can observe that these 137 drug compounds were clustered into four categories. The approved drugs CID000037542 and CID003010818 were in the same category ‘cluster0’. In this category, there were 20 other drug compounds, suggesting that these 20 drug compounds have a stronger association with the approved drugs than do other candidate compounds. Thus, they were deemed to be significant for HCV. The information of these 20 drug compounds are listed in **Table 1**.

**Table 1:** Information about the 20 significant candidate drug compounds for HCV.

Compound ID	Compound name	Determination value	Supporting references
CID000002022	Acyclovir	900	[26], [28]
CID000003454	Ganciclovir	499.5	[27], [28]
CID000216239	Sorafenib	710.08	[29], [30], [31]
CID000446155	Fluvastatin	691.57	[32], [33], [34]
CID000060734	Celgosivir	666.5	[35], [36], [37]
CID000051634	Miglustat	597	[38], [39], [40]
CID003062316	Dasatinib	556.63	[41], [42], [43]
CID000060822	Atorvastatin	674.42	[44]
CID000035370	Zidovudine	483.88	[45], [46]
CID000005901	6-azaurindine	292	[47], [48]
CID000159325	Torcetrapib	759.33	—a
CID003081361	Vandetanib	570.71	—
CID000024066	Zalcitabine	555.5	—
CID000123619	Etoricoxib	523	—
CID000148177	Perifosine	335.88	—
CID000006830	Guanosine Triphosphate	829.33	—
CID003052775	Rasagiline	550	—
CID009872939	SureCN3185172	499.5	—
CID000005291	Imatinib	490.46	—
CID011556711	Carfilzomib	442	—

a : no references can be presently found in the literature indicating that the corresponding compound possesses anti-HCV activities.

## Discussion

Three small-molecule drugs, including ribavirin (CID000037542), telaprevir (CID003010818) and boceprevir (CID010324367), were previously reported in DrugBank for the treatment of the hepatitis C virus [17], [18]. Except boceprevir without the interaction information concerning target genes of HCV, the determination values of ribavirin and telaprevir were 504.27 and 338, respectively. As mentioned in Section “Results of the method”, 270 drug compounds were obtained as candidate drugs for HCV. Then, according to the results of the randomization test, 135 drug compounds remained. To further filter false discoveries, these drug compounds, together with the approved drugs ribavirin (CID000037542) and telaprevir (CID003010818), were clustered by the EM algorithm. The clustering result showed that ribavirin and telaprevir were in the same category ‘cluster0’. According to the theory that “structurally similar molecules are likely to have similar properties” by Johnson *et al.* [25], we took the 20 compounds that belonged to ‘cluster0’ as candidates for further analysis and discussion. Detailed information about these 20 compounds is listed in **Table 1**.

Among these 20 compounds, half of them have been reported to have anti-HCV activities, as subsequently detailed in the following paragraphs.

**Acyclovir and Ganciclovir:** Acyclovir (CID000002022, see row 2 of **Table 1**) is an antiviral agent for the treatment and management of herpes zoster, genital herpes and chickenpox [26]. Ganciclovir (CID000003454, see row 3 of **Table 1**) is an analog of acyclovir and is used to treat complications of AIDS-associated cytomegalovirus infections [27]. These two drugs are also available for the treatment of chronic hepatitis C and are generally accepted by HCV medical specialists [28].

**Sorafenib:** Sorafenib (CID000216239, see row 4 of **Table 1**) is used for the treatment of advanced renal cell carcinoma and hepatocellular carcinoma [29]. Himmelsbach *et al.* reported that sorafenib effectively blocked HCV replication by inhibiting c-raf *in vitro* [30], so this drug cannot exert a synergetic effect with IFN-α, which activates c-raf to show anti-HCV activity [31].

**Fluvastatin:** Fluvastatin (CID000446155, see row 5 of **Table 1**) is used for the treatment of cardiovascular diseases and is also used as an adjunct to dietary therapy for preventing cardiovascular events [32]. Moreover, fluvastatin monotherapy was reported to modestly inhibit HCV replication in humans [33] and achieved a higher sustained virological response when combined with PegIFN-α and ribavirin compared with PegIFN-α/rabavirin [34].

**Celgosivir:** Celgosivir (CID000060734, see row 6 of **Table 1**) is an oral inhibitor of α-glucosidase I and interferes with HCV assembly by altering the host-directed glycosylation of the envelope proteins [35]. Durantel *et al.* reported that celgosivir monotherapy was well tolerated with a modest antiviral effect on HCV [36] and exerted a synergic effect with PegIFN-α/rabavirin in a phase II clinical trial [37].

**Miglustat:** Miglustat (CID000051634, see row 7 of **Table 1**) is used for the treatment of Gaucher's disease and has been approved for the treatment of progressive neurological symptoms in Niemann-Pick disease type C patients [38]. Additionally, a clinical trial using miglustat to treat HCV patients is ongoing [39], [40].

**Dasatinib:** Dasatinib (CID003062316, see row 8 of **Table 1**) is an oral tyrosine kinase inhibitor that has been approved for treating chronic myelogenous leukemia [41]. McCartney *et al.* reported that dasatinib showed anti-HCV activity by inhibiting HCV entry into hepatocytes but did not affect HCV RNA replication [42]. The IC_50_ values observed for dasatinib to inhibit HCVcc infection (infection with cell culture-derived HCV) and HCVpp entry (entry of HCV pseudoparticles), as calculated by Lupberger *et al.*, were 0.50±0.30 µM and 0.53±0.02 µM, respectively [43].

**Atorvastain:** Atorvastain (CID000060822, see row 9 of **Table 1**) is a HMG-CoA reductase inhibitor and exhibited a modest inhibitory effect on HCV RNA replication when combined with PegIFN [44].

**Zidovudine:** Zidovudine (CID000035370, see row 10 of **Table 1**) is a component of the antiretroviral combinations for treating HIV infections [45]. Vento *et al.* reported that zidovudine may remit HCV-induced chronic hepatitis in HIV-1-infected patients by inhibiting HCV replication or by improving the host immune response to HCV [46].

**6-azauridine:** 6-azaurindine (CID000005901, see row 11 of **Table 1**) is used as an antineoplastic antimetabolite by interfering with pyrimidine synthesis to inhibit the formation of nucleic acids [47]. Ueda *et al.* reported that 6-azauridine showed anti-HCV activity in an HCV drug assay system (ORL8), and the selective index from dividing the CC_50_ value by the EC_50_ value was 4.1 [48].

For the remaining 10 compounds listed in row 12–21 of **Table 1**, although we have not found any literature reporting their anti-HCV activities, the probability of these compounds having anti-HCV ability cannot be excluded. In the future, we will focus on expanding our knowledge of these compounds and further report their anti-HCV activities, if possible.

## Conclusions

This study proposed a new method to discover candidate drugs for HCV. Chemical-chemical interactions and chemical-protein interactions were used in this analysis, and the results showed that certain small-molecule drugs may have the capability to treat HCV. We hope that these findings may provide an alternative way to filter drug compounds with a low probability, thereby obtaining candidate drugs for the treatment of HCV and other diseases.

## Supporting Information

Table S1 List of 421 target genes of hepatitis C virus.
(PDF)

Table S2 List of chemical-protein interactions extracted in the second step of the method.
(PDF)

Table S3 List of determination values and p-values of 272 candidate drug compounds.
(PDF)

Table S4 List of clustering results of the 137 drug compounds by ‘EM’ in Weka.
(PDF)
