# Supplementary material for: Finding Candidate Drugs for Hepatitis C Based on Chemical-Chemical and Chemical-Protein Interactions
Source: PLoS One. 2014 Sep 16;9(9):e107767. doi: 10.1371/journal.pone.0107767 (PMC4166673; doi:10.1371/journal.pone.0107767)
Supplement: Table S1 — List of 421 target genes of hepatitis C virus. (PDF) [file pone.0107767.s001.pdf]

Table S1. 421 target genes of hepatitis C virus

ACP1	POU3F2	MLXIP	APOA1
AGRN	TRIM27	MORC4	APOE
APOA2	10-Sep	MORF4L1	ARFIP1
BCAR1	ACTN1	MVP	AXIN1
C1QBP	ACTN2	NAP1L1	BAX
CCNH	AEBP1	NAPL1L2	BIN1
CD68	ANKRD12	NCAN	C10orf30
CDKN1A	ANKRD28	NDC80	C9ORF6
CFL1	ARFIP2	NEFL	CADPS
COL4A2	ARHGEF6	NEFM	CADPS2
CREBBP	ARNT	NELL1	CCDC100
DDX3X	ARS2	NELL2	CCDC86
DDX3Y	ASXL1	NID1	CDK1
DDX5	B2M	NID2	CDK6
DICER1	BCAN	NOTCH1	CENPC1
EGFL7	BCKDK	N-PAC	CENTD2
EP300	BCL2A1	NUP62	CEP250
FADD	BCL6	OBSCN	CEP57
FAS	BZRAP1	PARP4	CEP63
FBLN2	C10orf18	PCYT2	CRABP1
FBLN5	C10orf6	PDE4DIP	CSK
FKBP7	C12orf41	PDLIM5	DNAJA3
FUNDC2	C14orf173	PICK1	FBL2
GAPDH	C16orf7	PKNOX1	FBXL2
GRN	C1orf165	PLEKHG4	FHL2
HBXAP	C1orf94	PNPLA8	FYN
HIVEP2	C9orf30	PRKACA	GOLGA2
HLA-A	CALCOCO2	PRM1	GPS2
HLA-E	CBY1	PRMT1	GRB2
HNRPK	CCDC21	PRMT5	GSK3A
HOXD8	CCDC37	PRRC1	GSK3B
HSPD1	CCDC52	PSMB8	HCK
JAK1	CCDC66	PSMB9	IGLL1
JAK2	CCDC95	PTBP2	IPO4
KRT18	CCHCR1	PTPRN2	ITGAL
KRT19	CD5L	RABEP1	LCK
KRT8	CDC23	RAI14	LIMS2
LPXN	CELSR2	RASAL2	LOC374395
LRRTM1	CEP152	RBM4	LYN
LTBP4	CEP192	RCN3	MAPK12
LTBR	CFP	RGNEF	MGC2574
MAGED1	CHPF	RICS	MGP

MEGF6	CORO1B	RINT1	MOBK1B
MMRN2	COX3	ROGDI	NAP1L2
NPM1	CSNK2B	RP11-130N24.1	NDRG1
NR4A1	CTGF	RSHL2	NFE2
PABPN1	CXorf45	RUSC2	NUCB1
PAK4	DEAF1	SBF1	OAS1
PLSCR1	DES	SDCCAG8	PARVG
PML	DLAT	SECISBP2	PDPK1
PSME3	DOCK7	SERPINF2	PIK3R1
RNF31	DPF1	SERPING1	PIK4CA
RXRA	DPP7	SERTAD1	PITX1
SETD2	EEF1A1	SESTD1	PMVK
SLC22A7	EFEMP1	SF3B2	PPP1R13L
SLC31A2	EFEMP2	SIAH1	PTMA
SMAD3	EIF1	SLIT1	RAF1
STAT1	EIF4ENIF1	SLIT2	RANBP5
STAT3	ERC1	SLIT3	RPL18A
TAF11	FAM120B	SMURF2	RRBP1
TATDN1	FAM65A	SNRPD1	SFRP4
TBP	FBF1	SNX4	SHARPIN
TNFRSF1A	FBLN1	SPOCK3	SMYD3
TP53	FBN1	SPON1	SORBS2
TP53BP2	FBN3	SRPX2	SORBS3
TP73	FES	SSX2IP	SRC
TSN	FIGNL1	STAB1	SRCAP
TXNL2	FLAD1	SVEP1	SSB
VIM	FLJ11286	SYNE1	TACSTD2
VWF	FN1	SYNPO2	TAF9
YWHAB	FRMPD4	TAF1	TGFBR1
YWHAE	FRS3	TBC1D2B	THBS1
YWHAZ	FTH1	TBK1	TMF1
YY1	FUCA2	TBXAS1	TRAF2
ZNF271	GAA	TGFB1I1	TRIOBP
CALR	GBP2	THAP1	UBASH3A
CANX	GFAP	TICAM1	USP19
CD209	GNB2	TMEM63B	VAPA
CLEC4M	GON4L	TRIM23	VAPB
HSPA5	HIST3H2BB	TRIO	VPS35
JUN	HIST4H4	TRIP11	VPS52
LTF	HOMER3	TXNDC11	ZH2C2
PFN1	HRMT1L2	UBE1C	ZNF646
TMSB4X	IKBKE	USHBP1	CEP68
CD81	IQWD1	UXT	CHUK
EIF2AK2	ITGB4	VCAN	EIF4A2

EIF2AK3	JAG2	XAB2	HAO1
ITGB1	KHDRBS1	XRN2	MGC2752
KIAA1411	KIAA1549	YY1AP1	NCL
LOC730765	KIF17	ZBTB1	OS9
PSMA6	KIF7	ZCCHC7	PKM2
SCARB1	KPNA1	ZHX3	PKN2
SDC2	L3MBTL3	ZMYM2	PPIB
SMEK2	LAMA5	ZNF281	TTC4
TF	LAMB2	ZNF410	TUBB2C
AGT	LAMC3	ZZZ3	FMNL1
AZGP1	LDB1	CREB3	FXYD6
CTSB	LOC728302	ELAC2	H19
MPDU1	LRRC7	TRAF3IP3	ISLR
RAB14	LRRCC1	UBQLN1	LMNB1
SERPINC1	LZTS2	CREBL1	MS4A6A
ST3GAL1	MAPK7	ACLY	NUP214
VTN	MBP	AHNAK	SSR4
ZG16	MEGF8	AHSA1	STRBP
C7	MLLT4	AKT1	UBQLN4
CIDEB			
