# Supplementary material for: Finding Candidate Drugs for Hepatitis C Based on Chemical-Chemical and Chemical-Protein Interactions
Source: PLoS One. 2014 Sep 16;9(9):e107767. doi: 10.1371/journal.pone.0107767 (PMC4166673; doi:10.1371/journal.pone.0107767)
Supplement: Table S2 — List of chemical-protein interactions extracted in the second step of the method. (PDF) [file pone.0107767.s002.pdf]

Table S2. 955 chemical-protein interactions extracted in the second step of the method.

Compound ID	Protein ID	Confidence score
CID000000174	ENSP00000228918	165
CID000000174	ENSP00000261597	157
CID000000174	ENSP00000329312	456
CID000000174	ENSP00000362616	165
CID000000174	ENSP00000368066	171
CID000000174	ENSP00000370007	211
CID000000546	ENSP00000374332	309
CID000002022	ENSP00000361666	900
CID000002130	ENSP00000263645	170
CID000002130	ENSP00000265368	231
CID000002130	ENSP00000305692	184
CID000003043	ENSP00000337839	330
CID000003324	ENSP00000225698	304
CID000003324	ENSP00000293288	160
CID000003414	ENSP00000337839	244
CID000003447	ENSP00000261693	178
CID000003447	ENSP00000263645	184
CID000003447	ENSP00000316228	292
CID000003447	ENSP00000329967	178

CID000003447	ENSP00000337825	150
CID000003447	ENSP00000342070	176
CID000003447	ENSP00000356087	209
CID000003447	ENSP00000356671	942
CID000003454	ENSP00000253408	199
CID000003454	ENSP00000361666	800
CID000004764	ENSP00000272065	163
CID000004778	ENSP00000228918	212
CID000004778	ENSP00000252486	151
CID000004778	ENSP00000261693	203
CID000004778	ENSP00000263645	211
CID000004778	ENSP00000264998	643
CID000004778	ENSP00000272065	563
CID000004778	ENSP00000313829	157
CID000004778	ENSP00000316228	328
CID000004778	ENSP00000337825	618
CID000004778	ENSP00000339007	322
CID000004778	ENSP00000349465	157
CID000004778	ENSP00000350941	700
CID000004778	ENSP00000357656	309
CID000005071	ENSP00000317327	174
CID000005291	ENSP00000220003	403

CID000005291	ENSP00000244741	162
CID000005291	ENSP00000251849	182
CID000005291	ENSP00000262477	198
CID000005291	ENSP00000264657	713
CID000005291	ENSP00000268058	253
CID000005291	ENSP00000269305	874
CID000005291	ENSP00000270202	984
CID000005291	ENSP00000274335	318
CID000005291	ENSP00000293288	214
CID000005291	ENSP00000332973	162
CID000005291	ENSP00000337825	975
CID000005291	ENSP00000339007	284
CID000005291	ENSP00000345494	843
CID000005291	ENSP00000346839	150
CID000005291	ENSP00000350153	189
CID000005291	ENSP00000350941	966
CID000005291	ENSP00000352400	973
CID000005291	ENSP00000357656	469
CID000005291	ENSP00000358360	208
CID000005291	ENSP00000360266	800
CID000005291	ENSP00000363071	631
CID000005291	ENSP00000364133	175

CID000005291	ENSP00000365012	654
CID000005291	ENSP00000370007	211
CID000005291	ENSP00000371067	761
CID000005311	ENSP00000216727	278
CID000005311	ENSP00000229239	193
CID000005311	ENSP00000233057	414
CID000005311	ENSP00000244741	564
CID000005311	ENSP00000251849	450
CID000005311	ENSP00000252486	150
CID000005311	ENSP00000253408	167
CID000005311	ENSP00000263253	281
CID000005311	ENSP00000264657	161
CID000005311	ENSP00000269305	715
CID000005311	ENSP00000270202	952
CID000005311	ENSP00000293288	750
CID000005311	ENSP00000307235	306
CID000005311	ENSP00000309591	233
CID000005311	ENSP00000315949	315
CID000005311	ENSP00000347979	916
CID000005311	ENSP00000349156	233
CID000005311	ENSP00000360266	859
CID000005311	ENSP00000364839	164

CID000005311	ENSP00000375736	180
CID000005360	ENSP00000270202	318
CID000005360	ENSP00000307046	443
CID000005360	ENSP00000339007	811
CID000005360	ENSP00000346839	842
CID000005360	ENSP00000349465	278
CID000005360	ENSP00000350153	189
CID000005360	ENSP00000350941	239
CID000005360	ENSP00000360266	162
CID000005360	ENSP00000395546	814
CID000005625	ENSP00000295566	186
CID000005625	ENSP00000321853	381
CID000005881	ENSP00000243050	209
CID000005881	ENSP00000261597	178
CID000005881	ENSP00000269305	824
CID000005881	ENSP00000270202	813
CID000005901	ENSP00000371067	292
CID000005939	ENSP00000263273	234
CID000005939	ENSP00000305692	364
CID000006021	ENSP00000233057	260
CID000006021	ENSP00000305692	462
CID000006021	ENSP00000350941	800

CID000006021	ENSP00000395546	220
CID000006021	ENSP00000415615	176
CID000006029	ENSP00000225698	164
CID000006029	ENSP00000233057	800
CID000006029	ENSP00000243776	425
CID000006029	ENSP00000293362	166
CID000006029	ENSP00000300413	488
CID000006029	ENSP00000318195	162
CID000006029	ENSP00000319169	183
CID000006029	ENSP00000343745	169
CID000006029	ENSP00000388526	348
CID000006245	ENSP00000265368	268
CID000006252	ENSP00000229239	714
CID000006252	ENSP00000253408	450
CID000006252	ENSP00000261597	150
CID000006252	ENSP00000269305	972
CID000006252	ENSP00000293288	710
CID000006252	ENSP00000296930	207
CID000006252	ENSP00000345656	313
CID000006252	ENSP00000346839	190
CID000006252	ENSP00000347979	824
CID000006252	ENSP00000349465	198

CID000006252	ENSP00000349977	395
CID000006252	ENSP00000360266	834
CID000006252	ENSP00000364839	176
CID000006252	ENSP00000371067	154
CID000006594	ENSP00000331504	176
CID000006594	ENSP00000368066	150
CID000006802	ENSP00000220003	174
CID000006802	ENSP00000251849	629
CID000006802	ENSP00000252486	180
CID000006802	ENSP00000253408	818
CID000006802	ENSP00000254584	201
CID000006802	ENSP00000257700	174
CID000006802	ENSP00000262320	167
CID000006802	ENSP00000262477	814
CID000006802	ENSP00000263273	230
CID000006802	ENSP00000264657	427
CID000006802	ENSP00000270202	830
CID000006802	ENSP00000274335	213
CID000006802	ENSP00000293288	169
CID000006802	ENSP00000296557	287
CID000006802	ENSP00000296930	162
CID000006802	ENSP00000300026	177

CID000006802	ENSP00000309503	188
CID000006802	ENSP00000311005	162
CID000006802	ENSP00000330054	525
CID000006802	ENSP00000337731	174
CID000006802	ENSP00000337825	163
CID000006802	ENSP00000339007	247
CID000006802	ENSP00000339299	164
CID000006802	ENSP00000343204	166
CID000006802	ENSP00000349465	241
CID000006802	ENSP00000350941	728
CID000006802	ENSP00000355537	179
CID000006802	ENSP00000360266	173
CID000006802	ENSP00000368678	164
CID000006802	ENSP00000370007	216
CID000006802	ENSP00000377941	177
CID000006802	ENSP00000395546	183
CID000006804	ENSP00000240123	172
CID000006804	ENSP00000247986	279
CID000006804	ENSP00000256897	900
CID000006804	ENSP00000284776	186
CID000006804	ENSP00000324173	186
CID000006804	ENSP00000330054	170

CID000006804	ENSP00000345494	180
CID000006804	ENSP00000350941	660
CID000006804	ENSP00000354394	418
CID000006804	ENSP00000359497	874
CID000006804	ENSP00000368678	168
CID000006830	ENSP00000035383	900
CID000006830	ENSP00000162330	900
CID000006830	ENSP00000215659	900
CID000006830	ENSP00000216479	188
CID000006830	ENSP00000217893	900
CID000006830	ENSP00000220003	905
CID000006830	ENSP00000222247	900
CID000006830	ENSP00000230354	909
CID000006830	ENSP00000250617	900
CID000006830	ENSP00000251849	993
CID000006830	ENSP00000256897	900
CID000006830	ENSP00000261597	900
CID000006830	ENSP00000270202	597
CID000006830	ENSP00000273739	900
CID000006830	ENSP00000274335	900
CID000006830	ENSP00000300161	900
CID000006830	ENSP00000330054	355

CID000006830	ENSP00000331504	904
CID000006830	ENSP00000337731	901
CID000006830	ENSP00000337825	906
CID000006830	ENSP00000346839	900
CID000006830	ENSP00000354633	900
CID000006830	ENSP00000355537	900
CID000006830	ENSP00000357656	904
CID000006830	ENSP00000362946	213
CID000006830	ENSP00000383205	900
CID000006830	ENSP00000395546	905
CID000006830	ENSP00000405229	900
CID000006830	ENSP00000406878	900
CID000006830	ENSP00000408012	900
CID000007361	ENSP00000261405	727
CID000007361	ENSP00000274335	164
CID000007361	ENSP00000337839	254
CID000008582	ENSP00000312946	233
CID000009223	ENSP00000228938	390
CID000009223	ENSP00000337731	218
CID000014982	ENSP00000395546	192
CID000014982	ENSP00000415615	192
CID000017957	ENSP00000225698	703

CID000017957	ENSP00000244741	163
CID000017957	ENSP00000260356	666
CID000017957	ENSP00000269305	944
CID000017957	ENSP00000299529	563
CID000017957	ENSP00000318861	216
CID000017957	ENSP00000332164	305
CID000024066	ENSP00000337839	284
CID000024066	ENSP00000361666	827
CID000024393	ENSP00000231751	540
CID000024393	ENSP00000264998	830
CID000024393	ENSP00000273550	690
CID000024393	ENSP00000318195	189
CID000024393	ENSP00000331504	322
CID000024861	ENSP00000228938	180
CID000024877	ENSP00000264998	534
CID000031401	ENSP00000236850	800
CID000031401	ENSP00000261693	165
CID000031401	ENSP00000293288	162
CID000031401	ENSP00000357156	165
CID000035370	ENSP00000269305	178
CID000035370	ENSP00000312436	714
CID000035370	ENSP00000324173	800

CID000035370	ENSP00000337839	171
CID000035370	ENSP00000340858	719
CID000035370	ENSP00000349465	198
CID000035370	ENSP00000361666	727
CID000035370	ENSP00000370201	364
CID000037542	ENSP00000233057	721
CID000037542	ENSP00000261693	362
CID000037542	ENSP00000263645	738
CID000037542	ENSP00000269305	800
CID000037542	ENSP00000316228	266
CID000037542	ENSP00000319977	657
CID000037542	ENSP00000320866	163
CID000037542	ENSP00000343204	187
CID000037542	ENSP00000347979	800
CID000037542	ENSP00000354394	669
CID000037542	ENSP00000388001	184
CID000038077	ENSP00000233057	170
CID000043860	ENSP00000270202	815
CID000051634	ENSP00000247461	677
CID000051634	ENSP00000263645	161
CID000051634	ENSP00000305692	953
CID000054445	ENSP00000002165	530

CID000054445	ENSP00000243776	169
CID000054445	ENSP00000247461	944
CID000054445	ENSP00000305692	720
CID000054445	ENSP00000320866	726
CID000054445	ENSP00000324173	857
CID000054445	ENSP00000340858	234
CID000054445	ENSP00000349252	155
CID000055709	ENSP00000340858	311
CID000055709	ENSP00000368678	199
CID000059226	ENSP00000231751	216
CID000060613	ENSP00000244741	809
CID000060613	ENSP00000253408	211
CID000060613	ENSP00000348273	260
CID000060652	ENSP00000263552	195
CID000060652	ENSP00000299529	255
CID000060652	ENSP00000419692	151
CID000060734	ENSP00000305692	862
CID000060734	ENSP00000349252	471
CID000060772	ENSP00000162749	237
CID000060772	ENSP00000233057	902
CID000060772	ENSP00000382840	199
CID000060795	ENSP00000321853	174

CID000060822	ENSP00000236850	992
CID000060822	ENSP00000243050	812
CID000060822	ENSP00000250092	800
CID000060822	ENSP00000252486	992
CID000060822	ENSP00000252597	222
CID000060822	ENSP00000260356	810
CID000060822	ENSP00000261693	360
CID000060822	ENSP00000270202	925
CID000060822	ENSP00000340858	169
CID000060822	ENSP00000355627	552
CID000060822	ENSP00000356954	823
CID000060822	ENSP00000356969	636
CID000060834	ENSP00000231751	190
CID000060843	ENSP00000270202	284
CID000060846	ENSP00000300161	290
CID000060846	ENSP00000355627	835
CID000060846	ENSP00000374332	225
CID000060846	ENSP00000416097	152
CID000060847	ENSP00000321853	642
CID000060866	ENSP00000053867	258
CID000060866	ENSP00000229239	202
CID000060866	ENSP00000340019	345

CID000060866	ENSP00000388526	248
CID000060871	ENSP00000231751	192
CID000060955	ENSP00000262320	174
CID000064147	ENSP00000231751	156
CID000064627	ENSP00000293362	260
CID000064627	ENSP00000361666	338
CID000064973	ENSP00000355627	188
CID000064989	ENSP00000347979	161
CID000065948	ENSP00000252486	162
CID000065948	ENSP00000264657	165
CID000065948	ENSP00000355627	517
CID000068368	ENSP00000348273	186
CID000068368	ENSP00000395546	475
CID000068368	ENSP00000415615	387
CID000071237	ENSP00000231751	192
CID000071237	ENSP00000236850	156
CID000072187	ENSP00000276692	228
CID000072402	ENSP00000337445	336
CID000072828	ENSP00000318195	156
CID000072968	ENSP00000265368	343
CID000073087	ENSP00000315112	178
CID000073087	ENSP00000364839	362

CID000073124	ENSP00000267953	230
CID000077993	ENSP00000231751	374
CID000082146	ENSP00000299529	258
CID000082146	ENSP00000419692	992
CID000091966	ENSP00000331504	283
CID000093860	ENSP00000233057	817
CID000093860	ENSP00000247668	174
CID000093860	ENSP00000256897	268
CID000093860	ENSP00000261479	165
CID000093860	ENSP00000264657	939
CID000093860	ENSP00000265734	152
CID000093860	ENSP00000269305	994
CID000093860	ENSP00000270202	753
CID000093860	ENSP00000293288	994
CID000093860	ENSP00000293308	204
CID000093860	ENSP00000293362	165
CID000093860	ENSP00000301838	423
CID000093860	ENSP00000307235	682
CID000093860	ENSP00000315112	167
CID000093860	ENSP00000324173	922
CID000093860	ENSP00000331310	187
CID000093860	ENSP00000342070	800

CID000093860	ENSP00000343204	225
CID000093860	ENSP00000359424	235
CID000093860	ENSP00000360266	991
CID000093860	ENSP00000363993	165
CID000093860	ENSP00000364016	165
CID000093860	ENSP00000371067	397
CID000093860	ENSP00000372595	165
CID000093860	ENSP00000372721	165
CID000093860	ENSP00000381698	268
CID000093860	ENSP00000393744	165
CID000093860	ENSP00000396813	433
CID000093860	ENSP00000402406	165
CID000093860	ENSP00000406797	573
CID000093860	ENSP00000406878	165
CID000093860	ENSP00000407233	165
CID000093860	ENSP00000407810	165
CID000093860	ENSP00000412027	165
CID000094635	ENSP00000343204	192
CID000100252	ENSP00000319977	167
CID000100665	ENSP00000265368	274
CID000100665	ENSP00000269305	201
CID000100665	ENSP00000296930	224

CID000100665	ENSP00000319977	496
CID000104741	ENSP00000162330	165
CID000104741	ENSP00000162749	810
CID000104741	ENSP00000220003	249
CID000104741	ENSP00000228938	150
CID000104741	ENSP00000243050	150
CID000104741	ENSP00000251849	256
CID000104741	ENSP00000260356	353
CID000104741	ENSP00000261597	343
CID000104741	ENSP00000265368	810
CID000104741	ENSP00000270202	729
CID000104741	ENSP00000293288	518
CID000104741	ENSP00000296930	200
CID000104741	ENSP00000300584	285
CID000104741	ENSP00000339007	913
CID000104741	ENSP00000340019	150
CID000104741	ENSP00000350941	671
CID000104741	ENSP00000354394	192
CID000104741	ENSP00000360266	690
CID000104741	ENSP00000367263	150
CID000104741	ENSP00000384917	326
CID000104762	ENSP00000340019	163

CID000104865	ENSP00000346839	159
CID000104865	ENSP00000356954	344
CID000107706	ENSP00000356671	223
CID000107918	ENSP00000216727	425
CID000107918	ENSP00000233057	729
CID000107918	ENSP00000248244	241
CID000107918	ENSP00000337445	195
CID000107918	ENSP00000340858	680
CID000107918	ENSP00000343204	425
CID000107918	ENSP00000345494	233
CID000107918	ENSP00000354394	691
CID000107918	ENSP00000388001	877
CID000108150	ENSP00000231751	167
CID000108150	ENSP00000261597	256
CID000108150	ENSP00000269305	159
CID000108150	ENSP00000360266	809
CID000108150	ENSP00000367545	278
CID000108150	ENSP00000374332	292
CID000108188	ENSP00000269305	254
CID000110635	ENSP00000231751	161
CID000119182	ENSP00000231751	232
CID000119182	ENSP00000339007	178

CID000119607	ENSP00000231751	269
CID000122108	ENSP00000319977	557
CID000122749	ENSP00000225698	292
CID000122749	ENSP00000269305	517
CID000122873	ENSP00000261597	280
CID000123146	ENSP00000265368	181
CID000123146	ENSP00000272065	289
CID000123146	ENSP00000293288	172
CID000123619	ENSP00000231751	873
CID000123619	ENSP00000264998	173
CID000123631	ENSP00000200181	155
CID000123631	ENSP00000244741	232
CID000123631	ENSP00000264657	604
CID000123631	ENSP00000265734	152
CID000123631	ENSP00000269305	325
CID000123631	ENSP00000270202	949
CID000123631	ENSP00000293288	209
CID000123631	ENSP00000307235	231
CID000123631	ENSP00000311005	383
CID000123631	ENSP00000337825	609
CID000123631	ENSP00000339007	395
CID000123631	ENSP00000350941	873

CID000123631	ENSP00000358510	343
CID000123631	ENSP00000360266	163
CID000123631	ENSP00000365012	640
CID000123631	ENSP00000367229	360
CID000123964	ENSP00000231751	152
CID000123964	ENSP00000320866	166
CID000124087	ENSP00000231751	167
CID000124087	ENSP00000337731	159
CID000124087	ENSP00000374069	176
CID000124088	ENSP00000320866	159
CID000124088	ENSP00000355627	170
CID000124092	ENSP00000162749	161
CID000124092	ENSP00000293288	829
CID000124092	ENSP00000293308	253
CID000126565	ENSP00000264657	872
CID000126565	ENSP00000371067	902
CID000130165	ENSP00000261405	288
CID000131682	ENSP00000231751	174
CID000132970	ENSP00000295566	524
CID000132999	ENSP00000231751	183
CID000134780	ENSP00000244741	825
CID000134780	ENSP00000265734	207

CID000134780	ENSP00000312436	150
CID000134780	ENSP00000364839	508
CID000134780	ENSP00000370007	211
CID000134780	ENSP00000371067	368
CID000146570	ENSP00000326381	281
CID000148121	ENSP00000232014	225
CID000148121	ENSP00000296930	201
CID000148177	ENSP00000231751	167
CID000148177	ENSP00000262320	184
CID000148177	ENSP00000269305	460
CID000148177	ENSP00000270202	989
CID000148177	ENSP00000293288	195
CID000148177	ENSP00000301838	173
CID000148177	ENSP00000331310	235
CID000148177	ENSP00000360266	284
CID000148192	ENSP00000253792	276
CID000148192	ENSP00000298925	233
CID000148192	ENSP00000356671	191
CID000148192	ENSP00000362095	154
CID000148192	ENSP00000366005	433
CID000148192	ENSP00000388526	769
CID000150311	ENSP00000231751	268

CID000150311	ENSP00000250092	163
CID000150311	ENSP00000252486	626
CID000150311	ENSP00000261693	736
CID000150311	ENSP00000357156	333
CID000151171	ENSP00000231751	180
CID000151193	ENSP00000270202	504
CID000151193	ENSP00000350941	180
CID000151193	ENSP00000370007	211
CID000153970	ENSP00000265734	315
CID000153970	ENSP00000296930	268
CID000153970	ENSP00000339007	216
CID000154256	ENSP00000220003	187
CID000154256	ENSP00000228938	150
CID000154256	ENSP00000356954	150
CID000157688	ENSP00000231751	172
CID000157688	ENSP00000267953	176
CID000158781	ENSP00000264657	226
CID000158781	ENSP00000346839	805
CID000158781	ENSP00000355627	686
CID000158781	ENSP00000356954	879
CID000159269	ENSP00000263273	159
CID000159324	ENSP00000231751	269

CID000159324	ENSP00000251849	870
CID000159324	ENSP00000264657	260
CID000159324	ENSP00000270202	484
CID000159325	ENSP00000236850	962
CID000159325	ENSP00000261693	490
CID000159325	ENSP00000356969	826
CID000159594	ENSP00000274335	161
CID000162010	ENSP00000231751	234
CID000170364	ENSP00000252486	222
CID000170364	ENSP00000262367	157
CID000170364	ENSP00000355627	199
CID000170364	ENSP00000419692	934
CID000176870	ENSP00000244741	165
CID000176870	ENSP00000264657	567
CID000176870	ENSP00000269305	499
CID000176870	ENSP00000270202	949
CID000176870	ENSP00000274335	151
CID000176870	ENSP00000307235	186
CID000176870	ENSP00000339007	243
CID000176870	ENSP00000344220	264
CID000176870	ENSP00000350941	342
CID000177358	ENSP00000269305	395

CID000177399	ENSP00000231751	206
CID000177399	ENSP00000337731	182
CID000178024	ENSP00000345008	178
CID000193962	ENSP00000339299	282
CID000208898	ENSP00000231751	207
CID000208908	ENSP00000225698	212
CID000208908	ENSP00000244741	177
CID000208908	ENSP00000270202	982
CID000208908	ENSP00000343745	151
CID000208908	ENSP00000368678	184
CID000216235	ENSP00000216479	353
CID000216235	ENSP00000231751	401
CID000216235	ENSP00000247461	165
CID000216235	ENSP00000309629	266
CID000216239	ENSP00000215659	351
CID000216239	ENSP00000251849	996
CID000216239	ENSP00000264657	926
CID000216239	ENSP00000270202	744
CID000216239	ENSP00000274335	181
CID000216239	ENSP00000293288	837
CID000216239	ENSP00000307235	687
CID000216239	ENSP00000337825	715

CID000216239	ENSP00000347979	904
CID000216239	ENSP00000350941	902
CID000216239	ENSP00000360266	809
CID000216239	ENSP00000365012	469
CID000216325	ENSP00000252486	297
CID000216325	ENSP00000261693	277
CID000216326	ENSP00000162749	150
CID000216326	ENSP00000244741	810
CID000216326	ENSP00000269305	281
CID000216326	ENSP00000270202	288
CID000216326	ENSP00000293288	162
CID000216326	ENSP00000339007	286
CID000216326	ENSP00000364133	150
CID000216326	ENSP00000371067	222
CID000216326	ENSP00000395546	277
CID000216326	ENSP00000415615	277
CID000216416	ENSP00000220003	182
CID000216468	ENSP00000386759	184
CID000219018	ENSP00000231751	279
CID000219018	ENSP00000236850	235
CID000219022	ENSP00000231751	297
CID000219022	ENSP00000236850	252

CID000285033	ENSP00000231751	212
CID000392622	ENSP00000252486	336
CID000392622	ENSP00000253408	167
CID000392622	ENSP00000261693	283
CID000392622	ENSP00000307046	225
CID000392622	ENSP00000309629	266
CID000392622	ENSP00000315477	247
CID000392622	ENSP00000343745	151
CID000392622	ENSP00000357156	244
CID000431963	ENSP00000305692	153
CID000441300	ENSP00000253408	150
CID000443040	ENSP00000265368	166
CID000444499	ENSP00000229239	228
CID000444499	ENSP00000263273	228
CID000444499	ENSP00000350941	274
CID000444818	ENSP00000162330	172
CID000444818	ENSP00000267953	193
CID000444818	ENSP00000360266	810
CID000445643	ENSP00000236850	800
CID000445643	ENSP00000243050	224
CID000445643	ENSP00000251849	179
CID000445643	ENSP00000264998	800

CID000445643	ENSP00000267953	304
CID000445643	ENSP00000269305	801
CID000445643	ENSP00000270202	818
CID000445643	ENSP00000300026	462
CID000445643	ENSP00000329312	232
CID000445643	ENSP00000349465	198
CID000445643	ENSP00000352400	944
CID000445643	ENSP00000360266	864
CID000445643	ENSP00000364133	488
CID000445643	ENSP00000388526	299
CID000445643	ENSP00000413152	650
CID000446155	ENSP00000236850	929
CID000446155	ENSP00000252486	945
CID000446155	ENSP00000260356	810
CID000446155	ENSP00000270202	888
CID000446155	ENSP00000355627	199
CID000446155	ENSP00000356954	367
CID000446155	ENSP00000356969	703
CID000446157	ENSP00000236850	700
CID000446157	ENSP00000252486	412
CID000446157	ENSP00000261693	150
CID000446157	ENSP00000356969	915

CID000446541	ENSP00000264335	226
CID000446541	ENSP00000270202	800
CID000446541	ENSP00000296930	165
CID000446541	ENSP00000309503	170
CID000446541	ENSP00000356954	274
CID000447715	ENSP00000346839	208
CID000448013	ENSP00000293288	401
CID000448013	ENSP00000349977	216
CID000448013	ENSP00000359424	187
CID000448013	ENSP00000386759	814
CID000451447	ENSP00000320866	162
CID000451668	ENSP00000224237	192
CID000451668	ENSP00000229239	191
CID000451668	ENSP00000231524	150
CID000451668	ENSP00000231751	201
CID000451668	ENSP00000260356	832
CID000451668	ENSP00000261366	150
CID000451668	ENSP00000267953	153
CID000451668	ENSP00000269305	982
CID000451668	ENSP00000270202	716
CID000451668	ENSP00000271332	284
CID000451668	ENSP00000273550	150

CID000451668	ENSP00000293288	150
CID000451668	ENSP00000296930	752
CID000451668	ENSP00000307156	154
CID000451668	ENSP00000307843	150
CID000451668	ENSP00000309629	196
CID000451668	ENSP00000315949	292
CID000451668	ENSP00000317902	150
CID000451668	ENSP00000319977	150
CID000451668	ENSP00000326888	408
CID000451668	ENSP00000331719	150
CID000451668	ENSP00000340858	150
CID000451668	ENSP00000351137	150
CID000451668	ENSP00000357656	194
CID000451668	ENSP00000360266	809
CID000451668	ENSP00000364839	297
CID000451668	ENSP00000365012	150
CID000451668	ENSP00000367545	700
CID000451668	ENSP00000371067	845
CID000451668	ENSP00000375736	180
CID000451668	ENSP00000378332	150
CID000457954	ENSP00000319977	298
CID000466151	ENSP00000053867	260

CID000476861	ENSP00000296930	286
CID000476861	ENSP00000339007	233
CID000476891	ENSP00000216286	370
CID000476891	ENSP00000264187	153
CID000476891	ENSP00000350941	670
CID000501640	ENSP00000247461	477
CID000501640	ENSP00000305692	865
CID000501640	ENSP00000320866	176
CID000501640	ENSP00000388526	171
CID000636380	ENSP00000356671	994
CID000667490	ENSP00000243050	427
CID000667490	ENSP00000293288	800
CID000969472	ENSP00000231751	283
CID001201426	ENSP00000261479	198
CID001201426	ENSP00000265368	174
CID002826718	ENSP00000231751	278
CID003000926	ENSP00000277541	235
CID003000926	ENSP00000305692	153
CID003010818	ENSP00000342070	338
CID003018352	ENSP00000230354	170
CID003018352	ENSP00000233057	161
CID003018352	ENSP00000318195	157

CID003018352	ENSP00000328169	222
CID003032583	ENSP00000263552	210
CID003036505	ENSP00000231751	270
CID003052775	ENSP00000229239	621
CID003052775	ENSP00000231751	191
CID003052775	ENSP00000293288	968
CID003052775	ENSP00000383623	420
CID003062316	ENSP00000215659	250
CID003062316	ENSP00000220003	224
CID003062316	ENSP00000224237	810
CID003062316	ENSP00000251849	616
CID003062316	ENSP00000264657	363
CID003062316	ENSP00000270202	698
CID003062316	ENSP00000307235	813
CID003062316	ENSP00000316779	181
CID003062316	ENSP00000329312	244
CID003062316	ENSP00000331504	292
CID003062316	ENSP00000337825	975
CID003062316	ENSP00000350941	998
CID003062316	ENSP00000352400	158
CID003062316	ENSP00000357656	979
CID003062316	ENSP00000365012	888

CID003062316	ENSP00000371067	417
CID003081349	ENSP00000355627	531
CID003081361	ENSP00000220003	458
CID003081361	ENSP00000270202	662
CID003081361	ENSP00000319977	161
CID003081361	ENSP00000337825	900
CID003081361	ENSP00000350941	960
CID003081361	ENSP00000357656	410
CID003081361	ENSP00000365012	444
CID003082555	ENSP00000231751	320
CID004369359	ENSP00000360635	222
CID004628825	ENSP00000296930	199
CID004628825	ENSP00000305692	199
CID005280352	ENSP00000260956	657
CID005280352	ENSP00000264998	651
CID005280352	ENSP00000349465	198
CID005280352	ENSP00000361666	186
CID005281078	ENSP00000349252	173
CID005281078	ENSP00000355627	498
CID005282451	ENSP00000236850	817
CID005282451	ENSP00000261693	250
CID005282451	ENSP00000264998	809

CID005282451	ENSP00000277541	809
CID005282451	ENSP00000357156	196
CID005288092	ENSP00000261366	206
CID005288092	ENSP00000261693	203
CID005288092	ENSP00000262367	159
CID005288092	ENSP00000263645	211
CID005288092	ENSP00000316228	328
CID005288092	ENSP00000329967	203
CID005288092	ENSP00000337825	165
CID005288092	ENSP00000356087	239
CID005289317	ENSP00000319977	724
CID005311027	ENSP00000356954	163
CID005312125	ENSP00000348273	183
CID005312128	ENSP00000263552	173
CID005327336	ENSP00000222330	715
CID005327336	ENSP00000233057	265
CID005327336	ENSP00000256897	206
CID005327336	ENSP00000265734	151
CID005327336	ENSP00000300413	205
CID005327336	ENSP00000324806	715
CID005327336	ENSP00000354218	183
CID005327336	ENSP00000395546	290

CID005327336	ENSP00000415615	290
CID005329099	ENSP00000270202	263
CID005352062	ENSP00000244741	538
CID005352062	ENSP00000251849	272
CID005352062	ENSP00000263253	306
CID005352062	ENSP00000268058	159
CID005352062	ENSP00000269305	919
CID005352062	ENSP00000293288	275
CID005352062	ENSP00000301838	258
CID005352062	ENSP00000347979	268
CID005352062	ENSP00000366005	180
CID005352062	ENSP00000375736	180
CID005481350	ENSP00000231751	156
CID005486971	ENSP00000231751	288
CID005493381	ENSP00000264998	270
CID005493381	ENSP00000293288	811
CID005493381	ENSP00000328169	191
CID005493381	ENSP00000386759	156
CID005493444	ENSP00000355627	727
CID006102725	ENSP00000375097	359
CID006433082	ENSP00000229239	222
CID006442177	ENSP00000262238	167

CID006442177	ENSP00000270202	949
CID006442177	ENSP00000274335	165
CID006442177	ENSP00000293288	201
CID006445540	ENSP00000231751	226
CID006445562	ENSP00000220003	510
CID006445562	ENSP00000270202	269
CID006445562	ENSP00000307235	308
CID006445562	ENSP00000337825	900
CID006445562	ENSP00000350941	859
CID006445562	ENSP00000357656	549
CID006445562	ENSP00000365012	510
CID006445562	ENSP00000371067	525
CID006473876	ENSP00000300026	548
CID006473876	ENSP00000352400	270
CID006476938	ENSP00000263645	385
CID006480442	ENSP00000231751	265
CID006505803	ENSP00000216479	319
CID006505803	ENSP00000232014	218
CID006505803	ENSP00000233057	192
CID006505803	ENSP00000251849	772
CID006505803	ENSP00000262435	282
CID006505803	ENSP00000264657	241

CID006505803	ENSP00000265734	508
CID006505803	ENSP00000269305	658
CID006505803	ENSP00000270202	756
CID006505803	ENSP00000273853	222
CID006505803	ENSP00000293288	481
CID006505803	ENSP00000295566	182
CID006505803	ENSP00000296930	857
CID006505803	ENSP00000315112	257
CID006505803	ENSP00000319169	433
CID006505803	ENSP00000350941	257
CID006505803	ENSP00000360266	201
CID006505803	ENSP00000364133	237
CID006505803	ENSP00000371067	284
CID006505803	ENSP00000373487	159
CID006509979	ENSP00000231751	385
CID006852123	ENSP00000262477	209
CID006852123	ENSP00000315112	249
CID006852123	ENSP00000340858	156
CID006916933	ENSP00000309629	247
CID006916933	ENSP00000348273	163
CID006916933	ENSP00000350941	332
CID006916933	ENSP00000359552	163

CID006918107	ENSP00000231751	306
CID006918155	ENSP00000231751	222
CID006918296	ENSP00000231751	193
CID006918456	ENSP00000356671	220
CID006918523	ENSP00000231751	220
CID006918523	ENSP00000370007	211
CID006918540	ENSP00000236850	502
CID006918540	ENSP00000261693	416
CID006918572	ENSP00000356954	174
CID006918572	ENSP00000360635	826
CID006918572	ENSP00000370201	203
CID006918638	ENSP00000269305	215
CID006918638	ENSP00000296930	201
CID006918638	ENSP00000318195	206
CID006918638	ENSP00000354394	151
CID006918638	ENSP00000356954	150
CID006918638	ENSP00000375736	161
CID009571836	ENSP00000269305	286
CID009577221	ENSP00000320866	161
CID009578005	ENSP00000231751	154
CID009604655	ENSP00000337731	231
CID009800306	ENSP00000299529	205

CID009800306	ENSP00000360266	923
CID009800306	ENSP00000419692	232
CID009803963	ENSP00000263253	273
CID009803963	ENSP00000264335	454
CID009803963	ENSP00000264657	176
CID009803963	ENSP00000270202	714
CID009803963	ENSP00000330054	204
CID009803963	ENSP00000332973	452
CID009803963	ENSP00000344220	242
CID009803963	ENSP00000355537	167
CID009803963	ENSP00000356969	359
CID009803963	ENSP00000364133	367
CID009809714	ENSP00000337825	800
CID009809714	ENSP00000350941	702
CID009829523	ENSP00000251849	181
CID009832423	ENSP00000261693	169
CID009841834	ENSP00000231751	154
CID009843749	ENSP00000277541	713
CID009865515	ENSP00000232014	250
CID009865515	ENSP00000243050	292
CID009865515	ENSP00000244741	206
CID009865515	ENSP00000370201	253

CID009865515	ENSP00000371067	156
CID009872939	ENSP00000251849	499
CID009872939	ENSP00000350941	500
CID009875401	ENSP00000356671	717
CID009875516	ENSP00000231751	218
CID009888590	ENSP00000262320	159
CID009909438	ENSP00000236850	592
CID009909438	ENSP00000356969	355
CID009913881	ENSP00000231751	180
CID009930048	ENSP00000231751	484
CID009930048	ENSP00000320866	183
CID009952884	ENSP00000231751	188
CID009952884	ENSP00000296930	281
CID009952884	ENSP00000339007	229
CID009955116	ENSP00000231751	253
CID009999276	ENSP00000053867	595
CID009999276	ENSP00000296930	186
CID009999276	ENSP00000321347	690
CID009999276	ENSP00000331310	611
CID010077129	ENSP00000364133	336
CID010116877	ENSP00000320866	284
CID010127622	ENSP00000242365	435

CID010127622	ENSP00000244741	157
CID010127622	ENSP00000251849	279
CID010127622	ENSP00000270202	705
CID010127622	ENSP00000296930	173
CID010127622	ENSP00000307235	395
CID010152654	ENSP00000231751	154
CID010182969	ENSP00000356671	511
CID010280735	ENSP00000320866	197
CID011234052	ENSP00000270202	212
CID011244031	ENSP00000231751	234
CID011244031	ENSP00000293288	180
CID011485656	ENSP00000222330	152
CID011485656	ENSP00000231751	154
CID011485656	ENSP00000264657	151
CID011485656	ENSP00000324806	152
CID011485656	ENSP00000354394	181
CID011513676	ENSP00000300026	668
CID011513676	ENSP00000307156	256
CID011556427	ENSP00000236850	226
CID011556427	ENSP00000261693	275
CID011556711	ENSP00000262238	274
CID011556711	ENSP00000406797	345

CID011556711	ENSP00000406878	707
CID011683005	ENSP00000340019	150
CID011960529	ENSP00000265368	224
CID015955413	ENSP00000342070	481
CID016131053	ENSP00000233057	218
CID016131053	ENSP00000247668	273
CID016131053	ENSP00000252486	227
CID016131053	ENSP00000261693	433
CID016131053	ENSP00000263645	794
CID016131053	ENSP00000316228	387
CID016131053	ENSP00000337825	207
CID016131053	ENSP00000339007	162
CID016131053	ENSP00000356954	205
CID016132446	ENSP00000305692	294
CID016139605	ENSP00000267953	284
CID016157882	ENSP00000270202	494
CID016157882	ENSP00000343204	812
CID016157882	ENSP00000355627	492
CID016157882	ENSP00000360266	809
CID016158207	ENSP00000231751	161
CID021944179	ENSP00000231751	442
CID021944179	ENSP00000320866	157

CID023724531	ENSP00000270202	302
CID023724859	ENSP00000224237	811
CID023724859	ENSP00000260356	159
CID023724873	ENSP00000231751	297
CID023724873	ENSP00000236850	252
CID023724978	ENSP00000342070	176
CID023725625	ENSP00000350941	183
CID024752837	ENSP00000372322	239
CID024832061	ENSP00000265734	365
CID024832061	ENSP00000293288	210
CID024832061	ENSP00000356954	163
CID024832061	ENSP00000359424	340
CID024838940	ENSP00000265340	198
CID024838940	ENSP00000305503	190
CID024838940	ENSP00000315477	216
CID024838940	ENSP00000316228	535
CID024838940	ENSP00000318165	201
CID024847756	ENSP00000344547	463
CID024847866	ENSP00000315477	196
CID024847866	ENSP00000316228	184
CID024848920	ENSP00000354045	192
CID024848920	ENSP00000360635	444

CID025181561	ENSP00000371067	347
CID044147092	ENSP00000243050	153
CID044201342	ENSP00000231751	165
CID044201343	ENSP00000278407	832
CID044421200	ENSP00000354394	180
CID044564107	ENSP00000320866	197
CID044588310	ENSP00000325527	309
CID044620969	ENSP00000253408	152
CID044620969	ENSP00000309503	267
CID044620969	ENSP00000410645	253
CID046897873	ENSP00000330054	205
CID049767348	ENSP00000352400	378
