# Supplementary material for: Finding Candidate Drugs for Hepatitis C Based on Chemical-Chemical and Chemical-Protein Interactions
Source: PLoS One. 2014 Sep 16;9(9):e107767. doi: 10.1371/journal.pone.0107767 (PMC4166673; doi:10.1371/journal.pone.0107767)
Supplement: Table S3 — List of determination values and p-values of 272 candidate drug compounds. (PDF) [file pone.0107767.s003.pdf]

Table S3. Determination values and p-values of 272 candidate drug compounds

Drug ID	Determination value	P-value
CID000000174	220.83	0.831
CID000000546	309	0.318
CID000002022	900	0.016
CID000002130	195	0.792
CID000003043	330	0.37
CID000003324	232	0.389
CID000003414	244	0.405
CID000003447	288.63	0.399
CID000003454	499.5	0.273
CID000004764	163	0.667
CID000004778	351.85	0.324
CID000005071	174	0.34
CID000005291	490.46	0.119
CID000005311	414.05	0.117
CID000005360	455.11	0.348
CID000005625	283.5	0.393
CID000005881	506	0.45
CID000005901	292	0.255
CID000005939	299	0.295

CID000006021	383.6	0.606
CID000006029	322.78	0.772
CID000006245	268	0.307
CID000006252	449.07	0.216
CID000006594	163	0.668
CID000006802	296.94	0.596
CID000006804	381.18	0.869
CID000006830	829.33	0.051
CID000007361	381.67	0.237
CID000008582	233	0.808
CID000009223	304	0.109
CID000014982	192	0.655
CID000017957	508.57	0.08
CID000024066	555.5	0.228
CID000024393	514.2	0.221
CID000024861	180	0.273
CID000024877	534	0
CID000031401	323	0.623
CID000035370	483.88	0.209
CID000037542	504.27	0.269
CID000038077	170	0.422

CID000043860	815	0
CID000051634	597	0.082
CID000054445	541.88	0.1
CID000055709	255	0.22
CID000059226	216	0.386
CID000060613	426.67	0.199
CID000060652	200.33	0.466
CID000060734	666.5	0.024
CID000060772	446	0.154
CID000060795	174	0.808
CID000060822	674.42	0.131
CID000060834	190	0.659
CID000060843	284	0.484
CID000060846	375.5	0.541
CID000060847	642	0.015
CID000060866	263.25	0.49
CID000060871	192	0.489
CID000060955	174	0.265
CID000064147	156	0.316
CID000064627	299	0.228
CID000064973	188	0.246

CID000064989	161	0.488
CID000065948	281.33	0.293
CID000068368	349.33	0.436
CID000071237	174	0.405
CID000072187	228	0.356
CID000072402	336	0.113
CID000072828	156	0.597
CID000072968	343	0.229
CID000073087	270	0.37
CID000073124	230	0.341
CID000077993	374	0.304
CID000082146	625	0.064
CID000091966	283	0.124
CID000093860	399.35	0.505
CID000094635	192	0.199
CID000100252	167	0.469
CID000100665	298.75	0.246
CID000104741	405.5	0.23
CID000104762	163	0.493
CID000104865	251.5	0.651
CID000107706	223	0.429

CID000107918	499.56	0.169
CID000108150	326.83	0.339
CID000108188	254	0.265
CID000110635	161	0.651
CID000119182	205	0.661
CID000119607	269	0.379
CID000122108	557	0.048
CID000122749	404.5	0.225
CID000122873	280	0.081
CID000123146	214	0.26
CID000123619	523	0.164
CID000123631	413.94	0.306
CID000123964	159	0.454
CID000124087	167.33	0.73
CID000124088	164.5	0.427
CID000124092	414.33	0.048
CID000126565	887	0.046
CID000130165	288	0.161
CID000131682	174	0.44
CID000132970	524	0.072
CID000132999	183	0.411

CID000134780	378.17	0.251
CID000146570	281	0.393
CID000148121	213	0.364
CID000148177	335.88	0.251
CID000148192	342.67	0.601
CID000150311	425.2	0.405
CID000151171	180	0.435
CID000151193	298.33	0.402
CID000153970	266.33	0.218
CID000154256	162.33	0.481
CID000157688	174	0.735
CID000158781	649	0.118
CID000159269	159	0.741
CID000159324	470.75	0.128
CID000159325	759.33	0.049
CID000159594	161	0.592
CID000162010	234	0.124
CID000170364	378	0.11
CID000176870	374	0.322
CID000177358	395	0.062
CID000177399	194	0.611

CID000178024	178	0.47
CID000193962	282	0.335
CID000208898	207	0.468
CID000208908	341.2	0.492
CID000216235	296.25	0.365
CID000216239	710.08	0.031
CID000216325	287	0.233
CID000216326	290.3	0.499
CID000216416	182	0.578
CID000216468	184	0.568
CID000219018	257	0.195
CID000219022	274.5	0.18
CID000285033	212	0.412
CID000392622	239.88	0.811
CID000431963	153	0.363
CID000441300	150	0.721
CID000443040	166	0.743
CID000444499	243.33	0.314
CID000444818	391.67	0.348
CID000445643	537.53	0.229
CID000446155	691.57	0.072

CID000446157	544.25	0.274
CID000446541	327	0.442
CID000447715	208	0.344
CID000448013	404.5	0.085
CID000451447	162	0.404
CID000451668	328.32	0.19
CID000457954	298	0.559
CID000466151	260	0.374
CID000476861	259.5	0.324
CID000476891	397.67	0.143
CID000501640	422.25	0.12
CID000636380	994	0
CID000667490	613.5	0.2
CID000969472	283	0.402
CID001201426	186	0.32
CID002826718	278	0.319
CID003000926	194	0.383
CID003010818	338	0.298
CID003018352	177.5	0.713
CID003032583	210	0.185
CID003036505	270	0.206

CID003052775	550	0.281
CID003062316	556.63	0.203
CID003081349	531	0.046
CID003081361	570.71	0.267
CID003082555	320	0.215
CID004369359	222	0.637
CID004628825	199	0.309
CID005280352	423	0.456
CID005281078	335.5	0.584
CID005282451	576.2	0.195
CID005288092	214.25	0.461
CID005289317	724	0
CID005311027	163	0.419
CID005312125	183	0.224
CID005312128	173	0.584
CID005327336	335.56	0.278
CID005329099	263	0.493
CID005352062	335.5	0.298
CID005481350	156	0.668
CID005486971	288	0.404
CID005493381	357	0.259

CID005493444	727	0.08
CID006102725	359	0.018
CID006433082	222	0.118
CID006442177	370.5	0.349
CID006445540	226	0.256
CID006445562	553.75	0.304
CID006473876	409	0.103
CID006476938	385	0
CID006480442	265	0.232
CID006505803	375.8	0.398
CID006509979	385	0.298
CID006852123	204.67	0.516
CID006916933	226.25	0.584
CID006918107	306	0.175
CID006918155	222	0.348
CID006918296	193	0.526
CID006918456	220	0.637
CID006918523	215.5	0.303
CID006918540	459	0.118
CID006918572	401	0.203
CID006918638	180.67	0.579

CID009571836	286	0.213
CID009577221	161	0.573
CID009578005	154	0.387
CID009604655	231	0.177
CID009800306	453.33	0.07
CID009803963	340.8	0.353
CID009809714	751	0.155
CID009829523	181	0.6
CID009832423	169	0.542
CID009841834	154	0.46
CID009843749	713	0
CID009865515	231.4	0.381
CID009872939	499.5	0.045
CID009875401	717	0.014
CID009875516	218	0.313
CID009888590	159	0.109
CID009909438	473.5	0.092
CID009913881	180	0.358
CID009930048	333.5	0.244
CID009952884	232.67	0.271
CID009955116	253	0.218

CID009999276	520.5	0.118
CID010077129	336	0.128
CID010116877	284	0.104
CID010127622	357.33	0.273
CID010152654	154	0.431
CID010182969	511	0.046
CID010280735	197	0.235
CID011234052	212	0.228
CID011244031	207	0.278
CID011485656	158	0.711
CID011513676	462	0.091
CID011556427	250.5	0.244
CID011556711	442	0.16
CID011683005	150	0.23
CID011960529	224	0.569
CID015955413	481	0
CID016131053	322.89	0.186
CID016132446	294	0.316
CID016139605	284	0.261
CID016157882	651.75	0.104
CID016158207	161	0.511

CID021944179	299.5	0.389
CID023724531	302	0.22
CID023724859	485	0.142
CID023724873	274.5	0.145
CID023724978	176	0.219
CID023725625	183	0.58
CID024752837	239	0.198
CID024832061	269.5	0.743
CID024838940	268	0.567
CID024847756	463	0.089
CID024847866	190	0.6
CID024848920	318	0.27
CID025181561	347	0.084
CID044147092	153	0.565
CID044201342	165	0.266
CID044201343	832	0
CID044421200	180	0.183
CID044564107	197	0.215
CID044588310	309	0.017
CID044620969	224	0.397
CID046897873	205	0.507

CID049767348	378	0.084
