# Supplementary material for: Finding Candidate Drugs for Hepatitis C Based on Chemical-Chemical and Chemical-Protein Interactions
Source: PLoS One. 2014 Sep 16;9(9):e107767. doi: 10.1371/journal.pone.0107767 (PMC4166673; doi:10.1371/journal.pone.0107767)
Supplement: Table S4 — List of clustering results of the 137 drug compounds by ‘EM’ in Weka. (PDF) [file pone.0107767.s004.pdf]

Table S4. Clustering results of the 137 drug compounds by 'EM' in Weka

Compound ID	Category
CID000002022	cluster0
CID000003454	cluster0
CID000005291	cluster0
CID000005311	cluster1
CID000005901	cluster0
CID000005939	cluster3
CID000006252	cluster1
CID000006830	cluster0
CID000007361	cluster3
CID000009223	cluster3
CID000017957	cluster1
CID000024066	cluster0
CID000024393	cluster1
CID000024861	cluster3
CID000024877	cluster1
CID000035370	cluster0
CID000037542	cluster0
CID000043860	cluster1
CID000051634	cluster0
CID000054445	cluster1

CID000055709	cluster3
CID000060613	cluster1
CID000060734	cluster0
CID000060772	cluster1
CID000060822	cluster0
CID000060847	cluster1
CID000060955	cluster3
CID000064627	cluster3
CID000064973	cluster3
CID000065948	cluster3
CID000072402	cluster3
CID000072968	cluster3
CID000082146	cluster2
CID000091966	cluster3
CID000094635	cluster3
CID000100665	cluster3
CID000104741	cluster1
CID000107918	cluster1
CID000108188	cluster3
CID000122108	cluster1
CID000122749	cluster1
CID000122873	cluster3

CID000123146	cluster3
CID000123619	cluster0
CID000124092	cluster1
CID000126565	cluster1
CID000130165	cluster3
CID000132970	cluster1
CID000134780	cluster1
CID000148177	cluster0
CID000153970	cluster3
CID000158781	cluster1
CID000159324	cluster1
CID000159325	cluster0
CID000162010	cluster3
CID000170364	cluster2
CID000177358	cluster3
CID000216239	cluster0
CID000216325	cluster3
CID000219018	cluster3
CID000219022	cluster3
CID000445643	cluster1
CID000446155	cluster0
CID000446157	cluster1

CID000448013	cluster1
CID000451668	cluster1
CID000476891	cluster3
CID000501640	cluster1
CID000636380	cluster2
CID000667490	cluster1
CID003010818	cluster0
CID003032583	cluster3
CID003036505	cluster3
CID003052775	cluster0
CID003062316	cluster0
CID003081349	cluster1
CID003081361	cluster0
CID003082555	cluster3
CID005282451	cluster1
CID005289317	cluster2
CID005312125	cluster3
CID005327336	cluster3
CID005352062	cluster3
CID005493381	cluster3
CID005493444	cluster1
CID006102725	cluster3

CID006433082	cluster3
CID006445540	cluster3
CID006473876	cluster1
CID006476938	cluster3
CID006480442	cluster3
CID006509979	cluster3
CID006918107	cluster3
CID006918540	cluster1
CID006918572	cluster2
CID009571836	cluster3
CID009604655	cluster3
CID009800306	cluster1
CID009809714	cluster2
CID009843749	cluster1
CID009872939	cluster0
CID009875401	cluster1
CID009888590	cluster3
CID009909438	cluster1
CID009930048	cluster3
CID009952884	cluster3
CID009955116	cluster3
CID009999276	cluster1

CID010077129	cluster3
CID010116877	cluster3
CID010127622	cluster3
CID010182969	cluster1
CID010280735	cluster3
CID011234052	cluster3
CID011244031	cluster3
CID011513676	cluster1
CID011556427	cluster3
CID011556711	cluster0
CID011683005	cluster1
CID015955413	cluster2
CID016131053	cluster3
CID016139605	cluster3
CID016157882	cluster1
CID023724531	cluster3
CID023724859	cluster1
CID023724873	cluster3
CID023724978	cluster3
CID024752837	cluster3
CID024847756	cluster1
CID024848920	cluster3

CID025181561	cluster3
CID044201342	cluster3
CID044201343	cluster1
CID044421200	cluster3
CID044564107	cluster3
CID044588310	cluster3
CID049767348	cluster3
